# Nanomaterial-mediated autophagy: coexisting hazard and health benefits in biomedicine

**DOI:** 10.1186/s12989-020-00372-0

**Published:** 2020-10-16

**Authors:** Xiaoli Feng, Yaqing Zhang, Chao Zhang, Xuan Lai, Yanli Zhang, Junrong Wu, Chen Hu, Longquan Shao

**Affiliations:** 1 Stomatological Hospital, Southern Medical University, 366 South Jiangnan Road, Guangzhou, 510280 China; 2 Nanfang Hospital, Southern Medical University, 1838 North Guangzhou Street, Guangzhou, 510515 China; 3 Orthodontic Department, Stomatological Hospital, Southern Medical University, 366 South Jiangnan Road, Guangzhou, 510280 China

**Keywords:** Nanomaterials, Autophagy, Physicochemical property, Hazard, Medical benefit, Pyroptosis, Non-coding RNAs, Anticancer, Neurodegenerative disease

## Abstract

**Background:**

Widespread biomedical applications of nanomaterials (NMs) bring about increased human exposure risk due to their unique physicochemical properties. Autophagy, which is of great importance for regulating the physiological or pathological activities of the body, has been reported to play a key role in NM-driven biological effects both in vivo and in vitro. The coexisting hazard and health benefits of NM-mediated autophagy in biomedicine are nonnegligible and require our particular concerns.

**Main body:**

We collected research on the toxic effects related to NM-mediated autophagy both in vivo and in vitro. Generally, NMs can be delivered into animal models through different administration routes, or internalized by cells through different uptake pathways, exerting varying degrees of damage in tissues, organs, cells, and organelles, eventually being deposited in or excreted from the body. In addition, other biological effects of NMs, such as oxidative stress, inflammation, necroptosis, pyroptosis, and ferroptosis, have been associated with autophagy and cooperate to regulate body activities. We therefore highlight that NM-mediated autophagy serves as a double-edged sword, which could be utilized in the treatment of certain diseases related to autophagy dysfunction, such as cancer, neurodegenerative disease, and cardiovascular disease. Challenges and suggestions for further investigations of NM-mediated autophagy are proposed with the purpose to improve their biosafety evaluation and facilitate their wide application. Databases such as PubMed and Web of Science were utilized to search for relevant literature, which included all published, Epub ahead of print, in-process, and non-indexed citations.

**Conclusion:**

In this review, we focus on the dual effect of NM-mediated autophagy in the biomedical field. It has become a trend to use the benefits of NM-mediated autophagy to treat clinical diseases such as cancer and neurodegenerative diseases. Understanding the regulatory mechanism of NM-mediated autophagy in biomedicine is also helpful for reducing the toxic effects of NMs as much as possible.

## Background

Nanomaterials (NMs) are defined as materials containing particles with one or more external dimensions in the size range from 1 to 100 nm, among which particulate NMs are also known as nanoparticles (NPs) [1]. Based on their unique physical and chemical properties, a growing number of studies have paid attention to their application prospects in the biomedical field to develop novel diagnostic or therapeutic tools, such as for drug delivery [2], biosensors [3], bioprobes [4], and tissue engineering materials [5]. Concurrent with wide nano applications are increasing exposure risks of NMs to the human body. In addition to environmental exposure, NMs and their composite systems can directly contact tissue or a damaged wound, as they are inhaled, taken orally, or even injected directly into the biological system in a medical situation [6–9]. For these considerations, a large number of studies have investigated the biological hazard of NMs through varying exposure pathways [10–13]. In view that NMs are more chemically active than their bulk counterparts, NMs distributed in different organs can cause distinct toxic effects through interacting with cells, proteins, or DNA after entering the body [14–17]. At present, the most widely investigated mechanisms of NM-driven toxicity include oxidative stress, inflammatory response and cell apoptosis, but other underlying mechanisms remain obscure and require our particular attention [18–20].

The definition of autophagy has been identified as an emerging mechanism of NM-induced toxicity in recent research. Autophagy (also called autophagic flux) is a conserved catabolic process by which impaired organelles and proteins are sequestered in double-membraned vesicles called autophagosomes [21]. In the initial step, the intracellular microtubule-associated protein light chain 3 (LC3) precursor is hydrolyzed by the autophagy-related protein 4 (Atg4) to generate water-soluble LC3-I, which is distributed in the cytoplasm. Subsequently, LC3-I covalently binds phosphatidyl ethanolamine (PE) under the synergistic action of Atg7 and the Atg12-Atg5-Atg16l complex to generate lipid-soluble LC3-II, which participates in autophagosome membrane extension [22]. Finally, the fusion of autophagosomes with lysosomes to form autolysosomes leads to the degradation of the encapsulated materials to their components, which can contribute to cell growth and maintain cell homeostasis. Autophagy can be activated as a protective mechanism under both physiological and pathological conditions to maintain or restore cell homeostasis [23]. Conversely, once out of balance following NM exposure, autophagy can also cause cell damage such as mitochondrial and lysosomal dysfunction, endoplasmic reticulum impairment, and even programmed cell death [24–27].

The biological behavior of NMs is regulated by multiple factors, among which autophagy not only initiates the upstream responses but also may be result from other biological effects [28]. For example, zinc oxide (ZnO) NPs and silica NPs (SiNPs) have been reported to disrupt the activity of antioxidant enzymes (catalase, glutathione peroxidase), resulting in intracellular oxidative stress imbalance and increased ROS levels, as well as activated excessive autophagy by inhibiting the PI3K/Akt/mTOR signaling pathway, leading to autophagic cell death [20, 29]. Moreover, normal autophagy, as an effective means for cells to resist foreign stimulation (such as NM exposure), can alleviate the apoptosis of Leydig TM3 cells [30]. However, when autophagic flux is blocked, such as autophagy-lysosome degradation functional declines following graphene oxide (GO) exposure, p62 protein as the substrate of autophagy accumulates substantially in the cell and even triggers apoptosis [31]. It is worth noting that increasing evidence indicates that new types of pathological changes, including pyroptosis, necroptosis, and ferroptosis, are also closely related to autophagy [32–34]. Combined with the above observations, autophagy may play different roles in the biological behavior of NMs, such as the promoter, transfer station, or effect endpoint, and this regulation mode may play completely opposite roles in physiological and pathological states. Studies in recent years have shown that autophagy can lead to the development of cancer [35], neurodegenerative disease [36], autoimmune disease [37], and cardiovascular disease [38]. Therefore, how to use NMs including their composites to regulate the autophagy process has very important reference value for the effective clinical treatment of these diseases.

This review first introduces the application of NMs in biomedical science based on different physicochemical properties and classifications. In view of the increasing exposure risk of NMs and their composites during the diagnosis and therapy process, we next describe their in vivo and in vitro toxic effects, which mainly focus on the regulation of NM-mediated autophagy. Moreover, the important role of autophagy in pathological conditions has been identified; thus, more attention should be paid to how to use NM-mediated autophagy as an effective therapeutic tool to promote the treatment of clinical diseases. We hope this review can help to understand the underlying mechanisms by which NMs regulate autophagy both in physiological and pathological states and further make better use of these advantages in the biomedical field.

### Search strategy

**Fig. 1:**
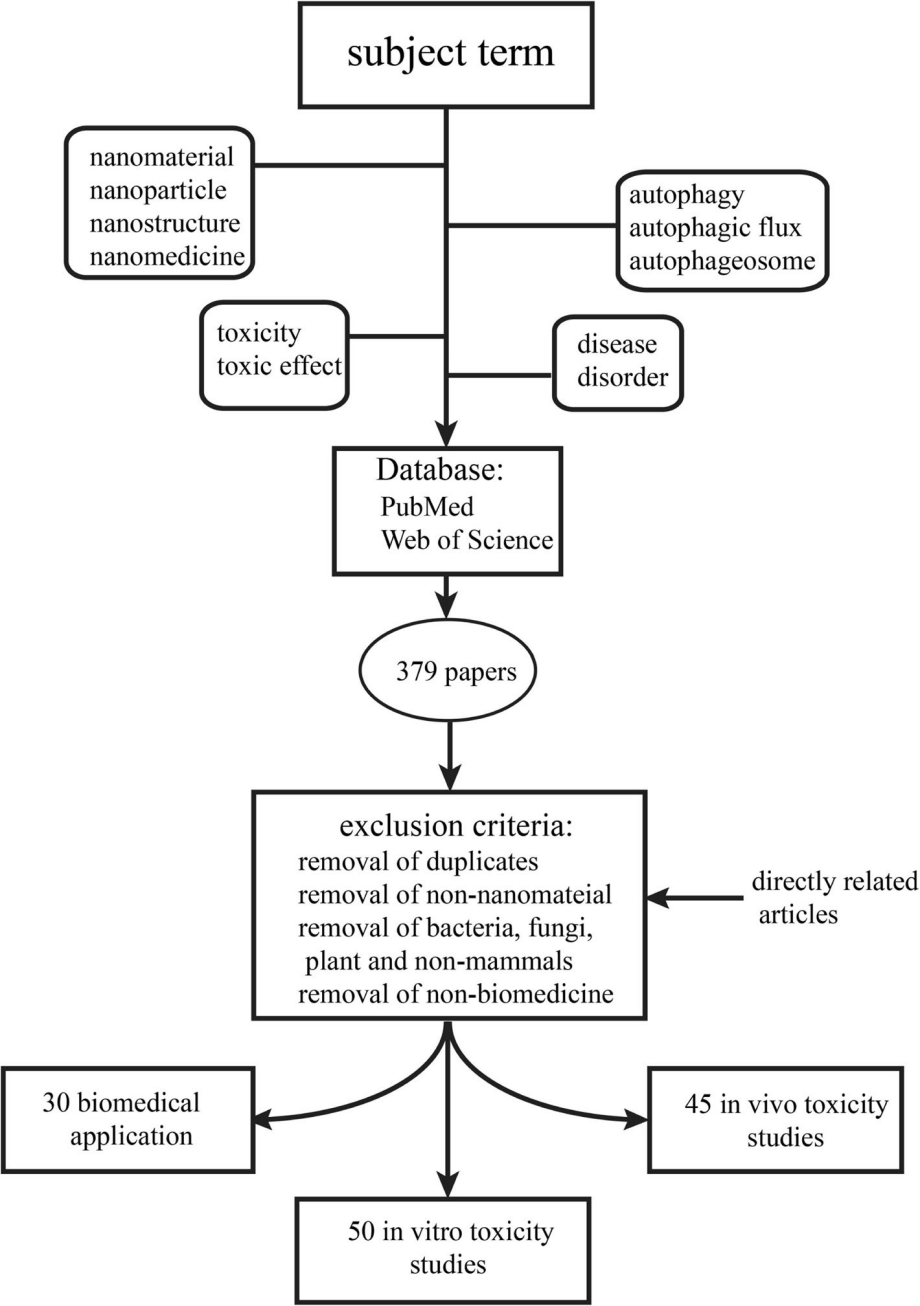
Literature search strategy. By searching the contents of four search boxes in two databases, 379 literatures were initially obtained. After refinement and exclusion, only the content directly related to this review was finally included (125 articles were obtained), which constituted the core literature of this paper. Moreover, other relevant literature is cited in appropriate places as a supplement

The search strategy of this paper is shown in Fig. 1. Databases such as PubMed and Web of Science were utilized to search the literature for relevant articles, which included all published, Epub ahead of print, in-process, and non-indexed citations. The purpose of this search was to look for papers related to autophagy mediated by NMs, not only in terms of its toxic effects but also biomedical treatments, such as those for cancer and neurological diseases. The retrieved articles were published from January 2009 to March 2020, and articles published in the past 5 years were preferentially chosen. Article types included research articles and reviews. The subject terms used in this search were as follows: (1) “nanomaterial” or “nanoparticle” or “nanostructure” or “nanomedicine” and (2) “autophagy” or “autophagic flux” or “autophagosome” and (3) “toxicity” or “toxic effect” and “diseases” or “disorder” (4) and (5) “biomedical application”. A total of 379 results were generated, and 125 directly related studies were obtained after elimination.

Exclusion criteria included the following:

- Editorials and commentaries;
- Research unrelated to NMs;
- Non-biomedical applications such as optoelectronic and mechanical applications;
- Studies involving bacteria, fungi, plants, and non-mammals such as shellfish and zebrafish;
- Duplicate references.

### Characteristics and applications of NMs in biomedicine

Nanomaterials have characteristics of a small size effect, high specific surface area, and quantum size effect, among others, which are obviously different from those of their bulk counterparts [39]. These effects largely determine their melting point, optical properties, chemical reactivity, magnetism, and superconductivity, contributing to their extensive application prospects in the field of biomedicine. A large number of studies have reported nano applications for the innovation of traditional diagnostic and treatment technologies, among which the most widely used NMs are listed in this review.

### Mesoporous silica NMs (MSNs)

Mesoporous silica mostly refers to amorphous silicon oxide materials with pores ranging from 2 to 50 nm. Mesoporous silicon-based NMs are composed of amorphous skeletons with a high pore capacity, are uniform with an adjustable pore size, and have an available functionalization and interface effect [40]. Furthermore, this kind of material has good biocompatibility, contributing to its wide application prospects in fields of biotechnology such as drug delivery [41] and gene therapy [42, 43]. The discovery of MSNs has greatly promoted the development of sustained and controlled drug release field. Conversely, we should note that MSNs can be degraded to a certain extent in vivo, but their degradation rate and degree are unpredictable and uncontrollable [44, 45]. How to adjust the degradation rate of MSNs to meet the clinical requirement, or reduce the potential risks introduced by its long-term survival in the human body by increasing their degradation level, is still the challenge of current research.

### Magnetic NMs

Functional NMs with a magnetic field response possess good modifiability, special in vivo metabolic behaviors, and unique magnetic properties, showing great application value in biological detection, disease diagnosis, and treatment. In recent years, a variety of functional composites based on iron oxide (Fe_2_O_3_) or ferrosoferric oxide (Fe_3_O_4_) NPs have been widely used in tumor molecular imaging and magnetic hyperthermia [46–48]. It is worth noting that many challenges persist in the clinical transformation of magnetic functional NMs. For example, the thermal conversion efficiency of magnetic NPs doped in magnetic hydrogel under magnetic field is low [49]. The high efficiency of the magneto-thermal medium will further weaken its biosecurity [50]. How to achieve low toxicity and high efficiency simultaneously is still the focus of future research.

### Metal NMs

Various morphologies of gold (Au) NMs have been reported in the literature, such as Au nanotubes [51], Au nanocages [52], hollow Au nanospheres [53] and Au nanorods [54]. Among them, Au nanorods are typical representatives. They have a plasma absorption peak with strong absorption in the near-infrared region [55], contributing to a good photothermal conversion efficiency [56]. Thus, they are often used in drug delivery and cancer treatment [57]. It should be noted that the shape of the NMs affects their intracellular uptake to a large degree. It has been reported that spherical AuNPs are more likely to enter cells than Au nanorods and cause downstream toxic effects [58]. In the preparation of multifunctional nano composites based on AuNPs, chemical reagents are often added as stabilizers (e.g., cetyl trimethylammonium bromide (CTAB)). However, CTAB not only hinders the coupling of Au nanorods with biomolecules [59], but also has certain biological toxicity. Silver (Ag) NPs are one of the most commonly used antibacterial materials. The antibacterial effects of AgNPs partly depend on their solubility. In the solution, AgNPs will release a high concentration of Ag ions, which can anchor the protein groups on the cell wall of bacteria and cause protein denaturation to form pores, eventually leading to increased permeability of the cell membrane and an imbalance of homeostasis [60]. In addition, studies have reported that AgNPs entering cells may directly damage DNA, RNA, and chromatin [61, 62]. These findings further lead to the consideration of the biocompatibility of AgNPs in clinical applications.

### Metal oxide NMs

ZnO NPs is often used in daily life as an additive in sunscreen products so that it can be in direct contact with human skin. In the field of biomedicine, it is widely used in biological imaging and antibacterial and anti-tumor applications [63–65]. Especially in the antibacterial field, it has a strong and broad-spectrum antibacterial effect on both gram-positive and gram-negative bacteria [66]. ZnO NPs are now considered to destroy lipids and proteins on the cell wall of bacteria, causing increased cell wall permeability and release of contents, finally leading to bacterial death. In addition, ZnO NPs can release free zinc ions in solution, which then produce reactive oxygen species (ROS) to kill bacterial cell bodies [67, 68]. Titanium dioxide (TiO_2_) is a kind of semiconductor material existing in nature, which has characteristics of stable chemical performance, easy availability at a cheap price, and high catalytic activity [69]. Nano-sized TiO_2_ has the physicochemical properties of a large specific surface area and small size simultaneously, which has attracted extensive attention in terms of photothermal treatment of tumors, surface modification of implants, and antibacterial agents [70–72]. At present, studies on the biological behaviors of ZnO and TiO_2_ NMs are relatively mature and comprehensive.

### Carbon-based NMs

Carbon-based NMs have shown great potential in drug delivery due to their unique characteristics of high specific surface area, good thermal conductivity, and easy surface modification. The hollow structures of fullerenes and carbon nanotubes (CNTs) could be used to carry drugs or other small molecules [73]. Large numbers of π electrons on the surface of graphene and CNTs can be used to load hydrophobic drugs [74, 75]. In addition, graphene and CNTs are also used as photothermal reagents for tumor photothermal therapy due to their strong near-infrared absorption capacity [76, 77]. Compared with other carbon-based NMs, the biocompatibility of the graphene family is relatively lacking. Although most in vivo studies have suggested that graphene-based NMs have good biological safety, there is still evidence indicating that they may cause oxidative stress by transferring electrons, leading to a series of toxic effects [78]. Conversely, tactics to improve the encapsulation efficiency of two-dimensional NMs including graphene and reduce the uncontrollable release of loaded drugs remains a challenge for future research.

Due to their wide applications in biomedical field, NMs and their composite systems can come in contact with normal tissue or a damaged wound, as they are inhaled, taken orally, or even injected directly into the biological system in medical situations. Considering that NMs are more chemically active than their bulk counterparts and can interact with biomolecules of similar small size such as proteins or DNA in vivo [15–17], it is important to rigorously investigate their biological behaviors.

### Distribution and metabolism of NMs in vivo

To simulate nano exposure under environmental or medical working conditions, different administration routes in animal models were used (summarized in Table 1), including airway exposure [79, 80], oral gavage [81], intravenous administration [82], intraperitoneal administration [83], and subcutaneous administration [84]. It should be noted that the administration route largely determines the distribution pattern and further target organ toxicity of NMs in the body. For example, intravenous and intraperitoneal injection of ZnO NPs leads to their deposition in the liver, spleen, and kidneys along with the blood circulation [85, 86], while nano-ZnO administered through intratracheal instillation mainly cause significant pulmonary inflammation in mice [87]. The physical and chemical properties are another important factor affecting the biological distribution of NMs. Larger GO aggregates are deposited near the injection site in Balb/c mice after intraperitoneal injection, while smaller aggregates are detected more easily absorbed and deposited in the proximity of the spleen serosa and liver [88]. In addition, a significant accumulation of polyethylene glycol (PEG) functionalized GO rather than pristine GO has been observed in the reticuloendothelial system (RES) after intraperitoneal injection, without obvious short-term toxicity [88], indicating a role of surface modification.

**Table 1:** In vivo toxicological studies of NPs mediated autophagy

NPs	Size (nm)	Coating	Animal model	Administration	Dose (mg/kg BW)	Exposure time	Organ toxicity	Autophagy alterations	References
TiO_2_ NPs	19.3 ± 5.4	bare	A/J Jms Slc mice (5 weeks old)	inhalation	2.5,5.0,10.0 (mg/m^3^)	6 h per day for 4 weeks	1. hyperplasia and hemorrhage 2. inflammatory response in the lung	induction of autophagy LC3 ↑ Beclin 1 ↑	[79]
Cd-based QDs	12	bare	male Balb/c mice	tail vein	0.1 ~ 0.3 nmol	24 h	1. increase of aspartate transaminase and glutamate pyruvate transaminase 2. haemocytes and necrosis in the live and kidney	stimulated autophagic flux LC3 ↑ P62 ↓	[80]
ZnO NPs	47.8	bare	Female Balb/c mice (8 weeks old)	Intraperitoneal or intravenous injection	10	once per week for 4 weeks	increase in serum creatinine and BUN in the kidney	autophagy induction autophagosome accumulation, LC3 ↑	[81]
	200–250	bare	Balb/c mice (6–8 weeks old)	oral gavage	200, 500	6 days	massive infiltration of inflammatory cells and DNA damage in the liver	autophagy induction autophagosome accumulation	[82]
UCNs	< 200	bare	male C57BL/6 J mice (6–8 weeks old)	tail vein	100	24 h	1. inflammatory cell infiltrates 2. ALT levels increased in the liver	induction of autophagy autophagosomes accumulation LC3 ↑ LAMP ↑	[83]
PAMAM NPs	5 ~ 6	bare	female Balb/c mice (6–8 weeks old)	intraperitoneal injection	100	10 days	1. hepatocytic necrosis and vacuolization 2. weight decreased 3. ALT and AST increased in the liver	induction of autophagy accumulation of vacuolization LC3 ↑	[84]
Graphene nanoplatelets	3 ~ 4	bare	ICR mice 6 Weeks old)	intratracheal instillation	2.5, 5	1, 7, 14, and 28 days	1. hyperplasia and hemorrhage 2. inflammatory response in the lung	blockade of autophaic flux LC3 ↑ P62 ↑	[85]
PAMAM NPs	_	bare	male Balb/c mice (6–10 weeks old)	intratracheal administration	50	24 h	lung inflammation and changed the lung elastance	induction of autophagy LC3 ↑	[86]
SWCNTs	–	COOH-CNT PABS-CNT PEG-CNT	male Balb/c mice(6–8 weeks old)	intratracheal administration	15	24 h	1. Acute pulmonary inflammation 2. severe lung edema	induction of autophagy autophagosomes accumulation LC3↑	[87]
CdTe QDs	4.08	bare	male Balb/c mice (8–10 weeks old)	intravenous injection	8 and 16 nmol/kg	24 h	1. increase in uric acid, creatinine and BUN 2. UPR and ER-phagy in the kidney and liver	induction of autophagy LC3↑	[88]
Fe_3_O_4_ NPs	15 ~ 20	PLGA	NIH mice	intraperitoneal injection	10	2, 4, 8, 10 and 12 days	extensive accumulation of autophagosome in the kidney and spleen	induction of autophagy autophagosomes accumulation LC3 ↑	[89]
MWCNTs	10 ~ 12	bare	male Wistar rats (200–220 g)	intraperitoneal injection	2.5	once per day for 14 days	decrease in hippocampal synaptic plasticity and spatial cognition in the brain	induction of autophagy LC3 ↑ Becline ↑	[90]
CoCr NPs	80 ± 14.6	bare	C57BL/6 mice (12 weeks old)	intravenous injection	0.12 mg per mouse	9.5, 12.5 days of pregnancy	1. increase in GFAP in hippocampus; 2. release IL-6 and DNA injury in the brain	blockade of autophaic flux LC3 ↑ P62 ↑	[91]
SiNPs	62	bare	Male and female ICR mice (8 weeks old)	intravenous injection	29.5, 103.5 and 177.5	14 ays	1.inhibitory effect on the expression of ICAM-1 and VCAM-1 2. impair angiogenesis	induction of autophagy LC3 ↑	[92]

The excretion of NMs is the final step of their metabolic kinetics in vivo, which is closely related with their distribution. For instance, intravenously and subcutaneously injected SiNPs accumulate in the liver and are primarily eliminated through hepatic processing and excreted via the biliary or feces route [89, 90]. Interestingly, Qi et al. [91] found that a majority of GO nanoplatelets (GONPs) and oxidized multiwalled carbon nanotubes (oMWCNTs) were excreted through feces after respective intravenous administration, while coexposure to both NMs led to urine excretion. This alteration was potentially due to chemical combination between NMs, an increased aggregation size, or the mutual influence of both mechanisms. Although the renal and fecal routes have been considered the main elimination routes for most NMs, the literature is insufficient to draw conclusions about their detailed clearance mechanism in vivo.

### Organ injury related to NM-driven autophagy

#### Hepatotoxicity

The liver is rich in lysosomes, and thus, under high levels of hunger stress, large amounts of autolysosomes can be produced and provide amino acids and glucose required for energy production of liver cells by degrading substrates to maintain normal physiological activities of the liver [92, 93]. Once autophagy is disrupted, the liver may show pathological changes such as viral hepatitis, or even the development of liver cancer [94–96]. NMs including AgNPs [97], rare earth upconversion NPs (UCNs) [98], and polyamidoamine dendrimers (PAMAM) NPs [99] have been reported to induce excessive autophagy to cause liver damage, characterized by hematoxylin and eosin (HE) staining in liver inflammatory cell infiltration, or dotted necrosis, and increased serum alanine aminotransferase (ALT) and aspartate aminotransferase (AST) activity. Moreover, the autophagy induced by SiNPs may further promote liver cell apoptosis through the accumulation of p62 protein and down-regulation of mammalian target of rapamycin (mTOR) [100].

#### Pulmonary toxicity

Environmental exposure to NMs significantly increases the possibility of entry into the human body through the respiratory tract and deposition mostly in the lungs. At present, a large amount of evidence indicates that NM-mediated autophagy is closely related to pulmonary toxicity [80, 101, 102]. For example, Park et al. [103] reported that the subchronic inflammatory response in the lungs caused by graphene nanoplatelets was associated with excessive autophagy, which suppressed ATP production and led to mitochondrial damage. Similar pulmonary injury was also detected in PAMAM NP and single-walled carbon nanotube (SWCNTs)-treated mice through Akt-TSC2-mTOR signaling [104, 105]. Interestingly, after carboxylation modification, the autophagy activity of multi-wall carbon nanotubes was significantly reduced, as manifested by the decreasing expression of LC3 [106, 107]. Moreover, the degree of carboxylation was negatively correlated with the pulmonary inflammatory response within a certain range, suggesting that the surface modification of NMs could help improve their biosafety. In addition to the effect of direct exposure, after maternal exposure to carbon black NPs during pregnancy, these NPs could cross the placental barrier and deposit in the lungs of offspring mice, leading to pulmonary fibrosis by inhibiting autophagy activation [108]. In our opinion, this finding highlights the importance and need for strict monitoring of women exposed to NMs and their composites during pregnancy.

#### Nephrotoxicity

Nanomaterials, especially those with a smaller particle size (<20 nm), are excreted mainly through the kidney in vivo [14]; thus, there is a great possibility that they will accumulate in the kidney and further cause adverse effects such as renal fibrosis or inflammation [109, 110]. Jiang et al. [111] found that intravenously injected cadmium telluride quantum dots were mostly distributed in the kidney of mice and triggered unfolded protein response (UPR)-mediated endoplasmic reticulum autophagy, leading to renal dysfunction. In addition, mice treated with ZnO NPs showed renal tubular dilatation and a flattened renal tubular epithelium, and serum uric acid and creatinine levels were also significantly increased. In combination with results from an in vitro study, the authors indicated that these findings were due to excessive autophagy activation through the hypoxia-inducible factor 1 (HIF-1) pathway following NP exposure [85]. This phenomenon was also confirmed in kidney exposed to Fe_3_O_4_ NPs, which showed other damage such as mitochondrial damage and ER stress. However, poly lactic-co-glycolic acid (PLGA) modification was found to reduce renal toxicity caused by pristine Fe_3_O_4_ NPs [112]. It should be noted that this finding was observed within 20 days, and PLGA may be degraded after long-term exposure to a biological environment (> 1 month); thus, it is still insufficient to draw conclusions regarding the long-term biosafety of PLGA-coated NPs in vivo.

#### Neurotoxicity

Autophagy plays a vital important role both in the maintenance of normal neural function but also the pathogenic mechanisms of the nervous system [113, 114]. It has been reported that nano alumina through carotid artery injection cross the blood-brain barrier to accumulate in the brain for one week, leading to local cerebral ischemia and even cerebral infarction [115]. Using the classic water maze experiment, Gao [116] found that intraperitoneal injected MWCNTs caused cognitive impairment in rats, which demonstrated decreased synaptic plasticity and hippocampal long-term potentiation (LTP). The addition of chloroquine (CQ), an autophagy inhibitor, significantly alleviated these lesions, suggesting that excessive activation of autophagy played an important role in MWCNT-induced neurotoxicity. In addition to direct exposure, maternal exposure to NMs during pregnancy might pose a safety risk to the health of offspring. Cobalt and chromium (CoCr) NPs injected via the jugular vein can reach the placenta barrier of mother rats, accumulate in the placenta, and disrupt the degradation function of autophagic flux, resulting in the release of inflammatory factor interleukin-6 (IL-6) and thus to DNA damage and abnormal differentiation of nerve progenitor cells in the brain of offspring mice [117].

#### Cardiovascular toxicity

By establishing exposed animal models through intratracheal instillation, inhalation, and skin contact, researchers have demonstrated that NM exposure causes cardiovascular toxicity [118–120]. It has been further reported that older rats [121] or rats with basic diseases such as hypertension [122] are more likely to develop pulmonary and cardiovascular lesions after exposure to NMs, suggesting that older people or populations with preexisting cardiovascular diseases are more sensitive to NM exposure and should be more closely monitored. At present, studies investigating the mechanism of NM-induced cardiovascular toxicity have mainly focused on the inflammatory response, oxidative stress, and mitochondrial damage [123–125]. There is evidence supporting that autophagy plays a role in regulating NM-driven cardiovascular toxicity [126]. After 14 days of intravenous injection, SiNPs resulted in increased levels of autophagosomes and LC3 and decreased expression of vascular endothelial growth factor receptor 2 (VEGFR2) in mouse heart tissue, suggesting an impaired angiogenesis capability. It was further suggested that the VEGFR2/PI3K/Akt/mTOR pathway regulated by SiNPs plays a leading role in autophagy activation and angiogenesis impairment. However, more in-depth studies are still required to investigate the regulatory mechanism of NM-mediated autophagy involved in the occurrence and development of cardiovascular diseases.

At present, a growing number of studies have reported that NMs can enter the brain via peripheral nerves such as the olfactory nerve through intranasal instillation [127–129]. Interestingly, there is new evidence supporting the transport of NMs through the taste nerve pathway into the brain, but the underling mechanism remains obscure [130]. With the widespread application of NMs in food processing, their oral exposure to the human body is increasing, and more in-depth studies are required to investigate the potential neurotoxicity of NMs acquired through the oral route. Additionally, most biosafety evaluations of NMs are limited to short-term (1–30 days) exposure, while the long-term (more than 30 days) toxic effect in the body remains largely unclear. We think that confirmation of the biosafety of NMs should be based on more primarily long-term in vivo studies, which can provide reliable scientific evidence for their clinical applications.

### In vitro toxicity

#### Cellular uptake

**Fig. 2:**
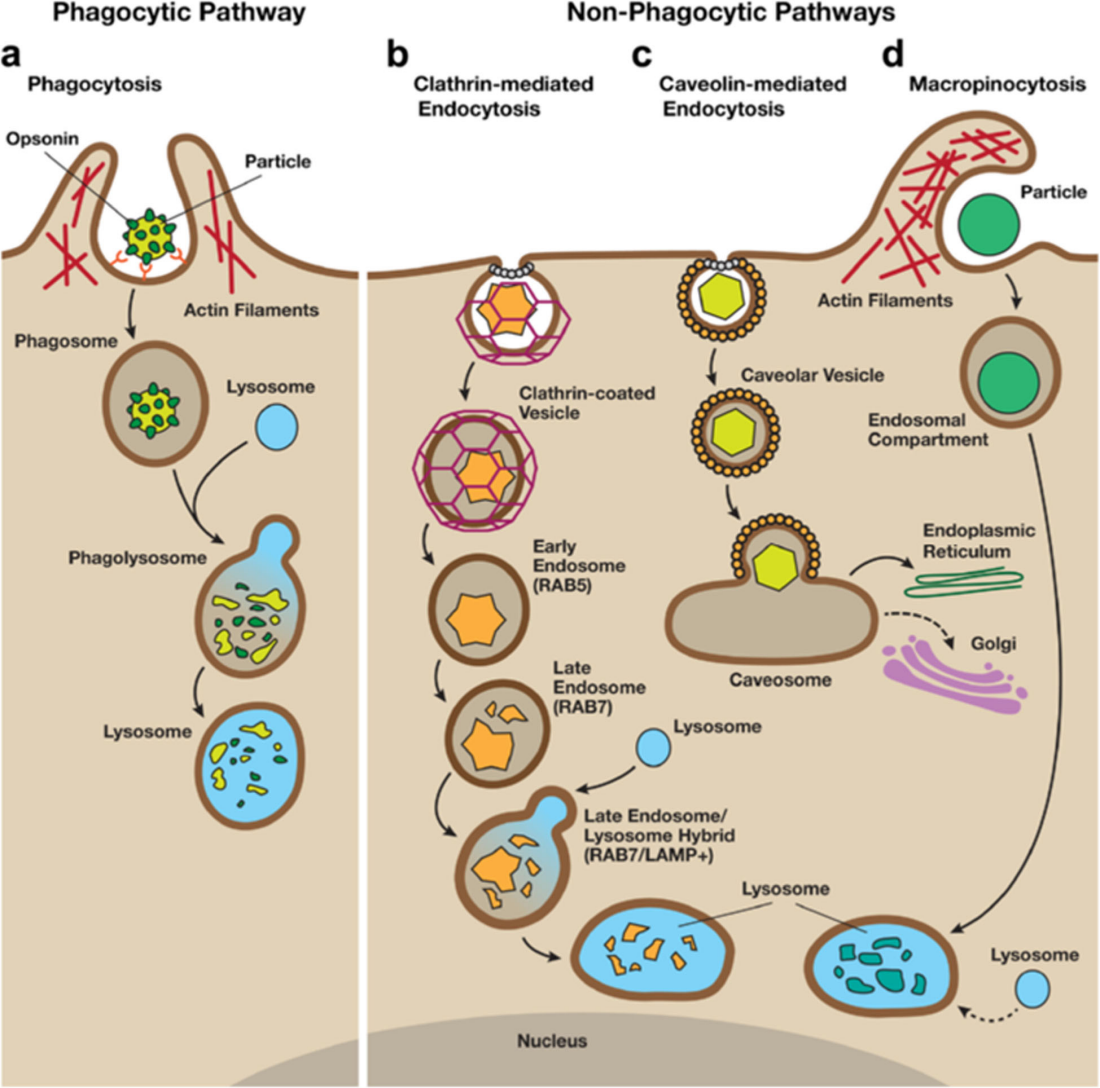
An overview of phagocytic and non-phagocytic pathways**. a** Phagocytosis occurs in macrophages through an actin-based mechanism involving interaction with various specialized cell surface receptors (such as mannose, IgG and complement receptors). The foreign particles recognized by specific receptors are internalized to form endocytic vesicles called phagosomes. The fusion of phagosomes with the lysosomal compartment leads to the formation of phagolysosomes, where the foreign particles are enzymatically degraded. **b** Clathrin-mediated endocytosis involves the formation of vesicles from triskelion clathrin-coated regions of the plasma membrane. After internalization, the clathrin are recycled back to the plasma membrane followed by movement of ingested materials from early endosome to late endosome, which finally fuse with lysosome to form the lysosome-endosome hybrid. The materials are then degraded by the low pH and enzyme-rich environment of the endo-lysosomal vesicle. C) Caveolin-mediated endocytosis involves internalization through caveolin (a dimeric protein) enriched invaginations. The cytosolic caveolin vesicle delivers its contents to endosomes to form caveosomes, which can transported along the cytoskeleton to the endoplasmic reticulum/golgi complex. D) Macropinocytosis involves the formation of large vesicles called macropinosomes, which occurs through actin filament driven plasma membrane protrusions. Through this pathway, the contents are degraded following fusion with the lysosomal compartment [28]. Reproduced with permission from Stern et al. (2012, BioMed Central) and had been partially adapted from Hillaireau and Couvreur (Cell. Mol. Life Sci. 2009, 66, 2873–2896)

NMs can be ingested by cells in different ways, which can be divided into phagocytic and nonphagocytic pathways (described in Fig. 2). Phagocytosis is a process by which specialized phagocytes engulf particles as large as 20 μm, suggesting a size-dependent behavior of NMs in the biological environment. Tabei et al. [131] revealed that the toxicity of MWCNTs in human promyelocytic leukemia cells (HL-60 cells) increased with enhanced phagocytic activity, which could be repressed by treatment with the phagocytosis inhibitor cytochalasin D. Unlike specialized phagocytic cells, nonphagocytic endocytosis occurs in all cells via four major mechanisms: clathrin-mediated endocytosis (CME), caveolae-mediated endocytosis (CvME), clathrin- and caveolae-independent endocytosis, and micropinocytosis [132]. After entering cells, NMs can interact with subcellular structures and trigger a series of biological effects by mediating autophagy [25, 133, 134].

#### The involvement of autophagy dysfunction in organelle impairment

Autophagy occurs and develops through a continuous dynamic process referred to as autophagic flux, which can be divided into five stages: initial, extension, autophagosome formation, autophagosome and lysosome fusion, and degradation in lysosomes [14]. Normal autophagy can remove abnormally synthesized proteins in cells and digest damaged and redundant organelles, which is beneficial for intracellular homeostasis. However, abnormalities in any of the above stages may lead to autophagic flux disorders and further disturb the physiological activity of the body [135, 136].

#### Mitochondrial disruption

In recent studies, mitochondrial disruption was found to be related to NM-mediated autophagy dysfunction, resulting in decreased cell viability, cellular dysfunction, or programmed cell death. For instance, fullerenol-induced mitochondrial dysfunction and cytotoxicity via excessive autophagy activation were partially alleviated by pretreatment with 3-methyladenine (3-MA), a classical autophagy inhibitor [24]. Conversely, GO-Ag NPs were detected to cause mitochondrial damage such as a decrease in mitochondrial membrane potential (MMP) in the first step, and further accelerate the progression of autophagy dysfunction [137]. Based on these results, the authors referred to mitophagy as a direct mechanism connecting mitochondrial disorder to (macro) autophagy, which can maintain mitochondrial homeostasis through clearance of damaged mitochondria and excessive superoxide anions [14]. Similar findings were also demonstrated in SiNP-treated human umbilical vein endothelial cells (HUVECs), which were manifested by decreased mitochondrial activity and membrane integrity that subsequently led to size-dependent mitophagy [138]. Mitophagy is a special type of macroautophagy in which the conditions of its occurrence and molecular regulatory mechanisms are closely related yet different from each other [98, 139]. Whether the occurrence and development of macroautophagy and mitophagy are consistent or whether these processes are mutually inhibitory remains to be further studied.

#### Lysosome dysfunction

Lysosomes contain various hydrolytic enzymes and are the most important organelles in the final stage of the autophagic process. Once normal lysosomal function is impaired, autophagic flux will be disturbed to a large extent. The three main toxic mechanisms of lysosome dysfunction are as follows: lysosomal ultrastructure impairment, lysosomal overload, and lysosome alkalization. First, lysosomal membrane permeabilization (LMP) has been found to be related to NM toxicity. For instance, Wang et al. [25] found that SiNPs could increase LMP to impair the lysosomal degradative function and further disturb downstream autophagic flux in hepatocytes. Similar toxicological manifestations were also demonstrated in CNT-treated human fibroblasts [140] and cationic polystyrene nanosphere-treated human macrophages [141]. Second, lysosomal overload may result from the excessive deposition of NMs and abnormal activities of lysosomal proteases [142, 143]. Copper oxide (CuO) NPs were prevalently deposited within lysosomes after cellular uptake, further leading to the impairment of autophagic flux by Cu^2+^ release in the lysosome, suggesting the important role of solubility in NP-induced toxicity [144]. Finally, lysosomal impairment is also reflected in the alkalization of lysosomes [145]. For example, larger (50 nm) AuNPs, not smaller (10 and 25 nm) particles, were more likely to be internalized by cells and lead to lysosome alkalization, which weakened the lysosome degradation capacity and ultimately blocked autophagic flux [146].

#### Endoplasmic reticulum impairment

The endoplasmic reticulum (ER), an integral and elaborate membrane organelle, has a powerful homeostatic system to achieve ER function that includes folding and modification of secretory proteins and synthesis of cholesterol and phospholipids [147]. However, when cells are stimulated by external factors (e.g., oxidative stress and ischemia reperfusion injury), ER homeostatic imbalance may occur, leading to endoplasmic reticulum stress (ERS) [148]. Furthermore, SiO_2_ NP-induced ERS can further activate autophagy to reduce ER damage, a process known as ER autophagy [149]. In contrast, compared with carboxylated polystyrene (COOH-PS), NH_2_-labeled polystyrene (NH_2_-PS) nanospheres exhibit a higher rate of cellular uptake to induce autophagy, ultimately leading to ERS and a decrease in cell activity [26]. Excessive stimulation at the initial stage of autophagy may lead to ER damage, and the blockade of autophagic flux can also accelerate the progression of ERS in magnetic iron oxide NP (M-FeNP)-treated macrophages, leading to cellular dysfunction and even cell death [133].

#### Cytoskeleton disruption

The cytoskeleton is an important structure by which eukaryotic cells maintain their basic morphology, including microtubules (MT), microfilaments (MF), and intermediate fibers (IF). A growing body of evidence has linked NM-induced cytoskeletal disruption with autophagy effects. Since the actin cytoskeleton participates in the formation and fusion of autophagosomes with lysosomes, any damage to the actin cytoskeleton can impair the above process, ultimately resulting in blockade of autophagic flux [126, 150]. Graphite carbon nanofibers (GCNF) can trigger apoptosis in human lung cells through excessive autophagosomes accumulation that resulted from cytoskeleton disruption-mediated autophagic flux blockade rather than autophagy induction [151]. Further evidence indicated that the impairment of the MT system, including MT clustering, fracture, and depolymerization, participated in ZnO NP-induced lysosome-autophagy system changes [152]. Therefore, MT acetylation was necessary for preventing damage to the MT structure and ensuring an intact pathway downstream of autophagic flux. The above two studies confirmed the important role of cytoskeleton disruption downstream of autophagy. However, does cytoskeleton damage affect processes upstream of autophagy, such as the extension and closure of double-layer membrane structures? Moreover, when autophagic flux is blocked and clearance capacity impaired, will excessive accumulation of abnormal MF and MT aggravate damage to the cytoskeleton system? Theses proposed questions still require in-depth investigations.

#### Golgi apparatus damage

The Golgi apparatus, a potential membrane source for autophagosome formation, is of great importance in the autophagy process. Among components of the Golgi apparatus, the seven-transmembrane, Golgi-resident protein PAQR3 has been demonstrated to function as a scaffold protein that facilitates the formation of autophagosomes [153]. However, little is known about the involvement of the Golgi apparatus in the regulation of NM-mediated autophagy, which may be due to the low probability of NM uptake by the Golgi apparatus. Interestingly, it was found that pleurotus tuber-regium (PTR)-conjugated selenium NPs (SeNPs) were distributed to the Golgi apparatus after internalization by human colon cancer cells (HCT 116 cells), and these NPs induced autophagy to promote cell apoptosis through upregulation of Beclin 1-related pathways [154]. However, whether the entry of NMs into Golgi bodies led to autophagy activation was not clarified in the study. We believe the above data suggest a functional link between the Golgi apparatus and NM-regulated autophagy, but more direct evidence is required.

#### DNA damage

In contrast to the above adverse effect resulting from NM-mediated autophagy, accumulating evidence suggests that autophagy is necessary for the repair process associated with the cellular response to DNA damage following NM simulation, which involves the AMPK or JNK signaling pathways [155, 156]. For example, cerium oxide (CeO_2_) NPs leads to the formation of DNA fragmentation followed by the activation of autophagy to restore DNA function, and the repair process was inhibited with the addition of 3-MA, which led to more severe DNA damage and even cell death [157]. It is worth noting that if DNA damage exceeds the repair capacity of autophagy, over-activated autophagy may aggravate the progression of DNA oxidative damage and even lead to cell death [134] .

In vitro toxicological studies of NM-mediated autophagy are summarized in Table 2. Currently, the literature is insufficient to draw conclusions about the cytotoxicity of NMs from different labs because of various factors, including the different physicochemical properties of NMs (e.g., size, functional group and solubility) and cell models. Furthermore, it is worth noting that autophagy may not only be a regulatory mechanism but also the result of other biological effects following NM exposure.

**Table 2:** In vitro toxicological studies of NPs mediated autophagy

NPs	Size (nm)	Coating	Cell line	Dose(ug/mL)	Uptake	Autophagy alteration	Biological effects	References
Fullerenol	2	bare	porcine renal proximal cells	0.1, 1, 10 nM	–	induction of autophagy autolysosome accumulation, LC3 ↑	mitochondrial membrane potential and ATP depletion, oxidative stress, cytoskeleton disruption, cell death	[24]
SiNPs	∼58.4 ± 7.4	bare	HepG2 cells	6.25, 12.5, 25, 50, 100	endocytose	induction of autophagy and blockade of autophagic flux autophagosome accumulation, LC3 ↑, P62 ↑	impaired the lysosomal function	[29]
GO	500–800	bare	PC12 cells	40,50,60	internalization	blockage of the autophagic flux, p62 ↑	Impairment of lysosomal degradation Apoptosis	[31]
CdTe-QDs	15	bare	HEK cells	15, 30, 60 nM	endocytosis	induction of autophagy, LC3 ↑	ER autophagy, unfolded protein response	[88]
Fe_3_O_4_ NPs	15 ~ 20	bare	MCF-7 cells	100	endocytosis	induction of autophagy autophagosome accumulation	impair the function of the lysosome, mitochondrial damage, ER and Golgi stress	[89]
GO-Ag NPs	15	bare	SH-SY5Y cells	5	–	induction of autophagy autophagosomes accumulation	mitichondrial dysfunction, ROS, DNA fragmentation, apoptosis	[137]
TiO_2_ NPs	15,50,100	bare	HeLa cells	10,50,100,500	internalization	blockage of the autophagic flux, LC3 ↑	lysosomal membrane permeabilization, α-synuclein accumulation	[139]
GQDs	3.28 ± 1.16	barre	GC-2 cells TM-4 cells	100	–	blockade of autophagic flux autophagosome accumulation p62 ↑	decreased the amount and enzymatic activity of cathepsin B, and inhibited lysosome proteolytic capacity	[142]
AuNPs	10, 25, 50	bare	NRK cells	1 nM	endocytosis	blockade of autophagic flux autophagosome accumulation, LC3 ↑, p62 ↑	Impairment of lysosome degradation capacity, Lysosome alkalinization	[146]
HA/β-Ga_2_O_3_:Cr^3+^ NPs	–	bare	SH-SY5Y cells	1,5, 25, 50	–	induction of autophagy, LC3 ↑, SQSTM/p62 ↑	ROS, calpain activation and neuronal damage	[150]
GCNs	outer diameter of 79 ± 6.6, inner diameter of 7 ± 0.8	bare	A549 cells	1, 10, 25, 50, 100	endocytosis	blockage of autophagic flux autolysosome accumulation	ROS, cytoskeleton disruption, apoptosis	[151]
SeNPs	80.0 ± 12.3 nm	bare	HCT 116 cells	2,5,10 μM	endocytosis	induction of autophagy, Beclin 1 ↑	cell cycle arrest, apoptosis	[154]
TiO_2_ NPs	22.07 ± 8.93	bare	BEAS-2B cells	6.25, 12.5, 25	internalization	induction of autophagy accumulation of autophagic vacuoles, LC3 ↑	overexpressed miR34a, mitochondrial dysfunction, cell death	[158]
PU NPs	64.3 ± 0.8	Carboxyl	macrophage cells	10,50	internalization	Induction of autophagy	Inhibition of inflammation and immune supression	[159]
SPIONs	60–80	bare	Raw264.7 cells BMDMs	10,20,50,100	endocytosis	Induction of autophagy autophagosomes accumulation, LC3 ↑, ATG5 ↑	Inflammatory responses	[160]
GO	MGO:1089.9 ± 135.3 SGO: 390.2 ± 51.4 NGO:65.5 ± 16.3 GQDs:5	bare	HUVECs	1,5,10,25	internalization	induction of autophagy, LC3 ↑ p62 ↓	Apoptosis, Apoptotic cell death, ER stress	[161]
	127 ± 4.7	PEG.	MCF7 cells MDA-MB-231 cells	4	–	inhibition of autophagy, LC3 ↓	apoptosis, suppressed Stathmin1 protein, decreases the microtubule instability, cancer cell death	[162]

### The interactions between autophagy and other biological effects

#### Oxidative stress and autophagy

The interactions of NMs with cells can lead to intracellular ROS generation, the intrinsic characteristics of which are the basic mechanisms essential for the growth or aging of organisms. However, when ROS levels exceed the activity of intracellular antioxidant enzymes, the resulting oxidative stress imbalance can induce cytotoxicity, such as apoptosis and DNA damage [18, 158, 159]. Furthermore, ROS-dependent autophagy is a widely accepted paradigm of autophagy induced by NMs. However, the role of NM-mediated autophagy is contrasting depending on different investigation models. Exposure of HUVECs to MSNPs triggered excessive autophagy via ROS-oxidative stress-mediated PI3K/Akt/mTOR signaling that could be reversed by pretreatment with the antioxidant N-acetyl L-cysteine (NAC), which was due to the successful activation of antioxidant enzymes [29]. Apart from treatment with antioxidants, reducing the solubility of paclitaxel NPs can decrease the production of ROS in tumor cells, thus inhibiting autophagic cell death [160]. ROS-mediated autophagic cell death was also demonstrated in SiNP-treated human hepatoma cells (HepG2 cells) and TiO_2_ NP-treated keratinocytes [161, 162]. Interestingly, other TiO_2_ NPs induced autophagy via the AMPK-mTOR signaling pathway as an antioxidant mechanism, protecting podocytes from oxidative damage and playing a pro-survival rather than a pro-death role [163]. In summary, oxidative stress may be the initial stage of toxicity of NMs, and considering that oxidative stress and autophagy regulate each other to form a positive feedback loop, an effective means to improve the biological safety of NMs is to regulate the autophagy level to maintain the oxidative stress balance.

#### Inflammation and autophagy

An increasing number of NMs have been demonstrated to induce significant inflammatory responses including inflammatory cell infiltration and granuloma formation [164]. Autophagy activation, as a protective mechanism against the dextran-coated Fe_3_O_4_ NP-induce inflammatory response, is accompanied by decreased levels of tumor necrosis factor-α (TNF α) and IL-1β [165]. Similarly, both curcumin-loaded selenium NPs (Se-Cur NPs) [166] and MSNs [167] are able to evoke autophagy and attenuate the inflammatory effect mediated by the NF-κB signaling pathway. Upon autophagy deactivation, polyurethane NPs (PU NPs) also reduces the death rate by inhibiting the recruitment of macrophages and monocytes [168]. Furthermore, unmodified Fe_3_O_4_ NPs can lead to endothelial dysfunction and inflammation by excessive autophagy induction that may be highly associated with the Beclin-1/VPS34 complex [19]. Superparamagnetic iron oxide NPs (SPIONs) trigger toll-like receptor-4 (TLR-4)-dependent autophagy, which promotes the phosphorylation of p38 and nuclear translocation of Nrf2, finally leading to the upregulation of p62/SQSTM and inflammatory response [169]. NP-induced autophagy is crucial for NP metabolism and cytotoxicity, but there have been insufficient studies on how the host immune system responds to NP-induced autophagy. Considering that certain inflammatory diseases such as psoriasis and lamellar ichthyosis are associated with autophagy [170], it is necessary to identify more molecular targets of autophagy and inflammation and use characteristics of NM-regulated autophagy to treat inflammation-related diseases.

#### Apoptosis and autophagy

Apoptosis, as a type of programmed cell death, features cell shrinkage, membrane blebbing, internucleosomal DNA fragmentation, chromatin condensation, and the formation of apoptotic bodies [171]. The crosstalk between apoptosis and autophagy has been confirmed in various studies of NMs. For example, ZnO NPs cause excessive autophagy via inhibiting the PI3K/Akt/mTOR signaling pathway, leading to cell apoptosis manifested by the cleavage of the apoptosis markers caspase 3, caspase 8, and caspase 9 [20]. CeO_2_ NPs can trigger autophagy at relatively low doses (5 or 10 μg/mL), and the subsequent activation and relocation of Bax to the mitochondria were followed by mitochondrial-dependent apoptosis [27]. Additionally, our previous research has demonstrated that GO can impair autophagic flux through the alkalization of lysosomes, eventually resulting in p62/SQSTM-dependent apoptosis, as confirmed by caspase 3/9 activation [31]. GO was also shown to induce autophagy via c-Jun N-terminal kinase (JNK)-mediated phosphorylation of B-cell lymphoma 2 (Bcl-2), accompanied by the dissociation of the Beclin-1/Bcl-2 complex, prompting the execution of apoptosis [172]. In conclusion, the initiation of autophagy leads to the inactivation of antiapoptotic proteins, and autophagic flux impairment leads to the accumulation of autophagy substrates that primarily trigger apoptosis directly mediated by the endogenous mitochondrial pathway, suggesting that mitochondria are the pivotal intermediate link between autophagy and apoptotic effects.

#### Necroptosis and autophagy

Necroptosis, which is known as “programmed necrosis”, is critically regulated by receptor interaction protein kinase 1 and 3 (RIP1 and RIP3 respectively) and plays an important role in immune system regulation, tissue injury, and cancer development [173–175]. Furthermore, autophagy and necroptosis may influence each other and form a positive feedback loop, promoting the process of cell death in nano-toxicology studies. The anticancer agent graphene oxide-chloroquine (GO-CQ) nanoconjugate was designed to induce the accumulation of autophagosomes through the blockade of autophagic flux, which served as a scaffold for necrosome assembly in human lung adenocarcinoma cells (A549 cells), leading to cell necroptosis [33]. In turn, hyaluronic acid-modified NPs loaded with CQ significantly upregulated RIP3 expression, subsequently interrupting autophagic flux via LMP, leading to autophagic cell death [25]. Little is known about the regulatory mechanism between autophagy and necroptosis, but autophagosomes may be upstream or downstream targets of the RIP1/RIP3 complex. Additionally, functionalized NMs can be designed as an effective therapeutic agent by their targeting of the autophagy-necroptosis axis.

#### Epigenetic alteration and autophagy

Epigenetic phenomena are numerous, including DNA methylation, genomic imprinting, maternal effects, gene silencing, nucleolus dominance, dormant transposon activation, and RNA editing [176]. MicroRNAs (miRNAs), a form of non-coding RNA, can regulate autophagy effects involved in the occurrence and development of cancer. Based on the unique physical and chemical properties of NMs, the present research focused on how to combine nanocarriers to improve the biological stability of miRNAs because they are susceptible to acidic degradation and deactivation inside endosomes and lysosomes [177, 178]. The cationic GO nanoplatform, an efficient targeted delivery system for the transfection of human breast cancer cells (MCF7 cells) with miR-101, has been shown to downregulate autophagy and subsequently contributes to 68% apoptosis [179]. Conversely, chitosan NPs used as carriers for miRNA 34a inhibit the growth of prostate cancer cells by inducing noncanonical autophagy via a proapoptotic effect [180].

DNA methylation is one of the most important epigenetic modulators, alterations of which are attracting growing attention as an underlying molecular target of NMs toxicity [181, 182]. In addition, DNA methylation may upregulate cell autophagy to reduce the sensitivity of hepatoma cells to chemotherapy or promote the development of diabetes due to the deacetylation of autophagy-related proteins [183, 184]. Given that DNA methylation is associated with the above diseases, further study of the relationship between NMs and DNA methylation provides a new way to evaluate the biocompatibility of NMs. Moreover, autophagy may act as a downstream mechanism of epigenetic changes and work together to regulate cell survival to promote the effects of nanotherapy.

#### Others

Pyroptosis is a type of proinflammatory programmed cell death featuring gasdermin family-mediated membrane pore formation and cell lysis, accompanied by the release of proinflammatory contents [185]. Various NMs have been shown to cause pyroptosis, mainly regulated by caspase-1 signaling pathways [186–188]. Molecules upstream of the caspase-1 signaling pathway involve the NLRP3 inflammatory complex, including cathepsin B, mitochondrial reactive oxygen species (mtROS), and mitochondrial DNA (mtDNA) [189, 190]. Conversely, strong autophagy induced by adrenomedullin via the ROS-AMPK-mTOR signaling pathway can alleviate the pyroptosis of Leydig cells; similar effects were observed for galangin-treated glioblastoma multiforme, suggesting that autophagy may initially serve as a protective mechanism to prevent pyroptosis [191, 192]. Studies on the relationship between autophagy mediated by NMs and pyroptosis are insufficient. As mitophagy dysfunction is related to the generation of mtROS and mtDNA [193], which can trigger caspase1-dependent pyroptosis [188, 189], we speculate that the mitophagy dysfunction caused by NMs may be associated with pyroptosis, meriting further study. Therefore, the use of NMs to activate autophagy may be an efficient strategy to treat clinical diseases related to pyroptosis, such as diabetic cardiomyopathy, infectious diseases, and atherosclerosis [194–196].

**Fig. 3:**
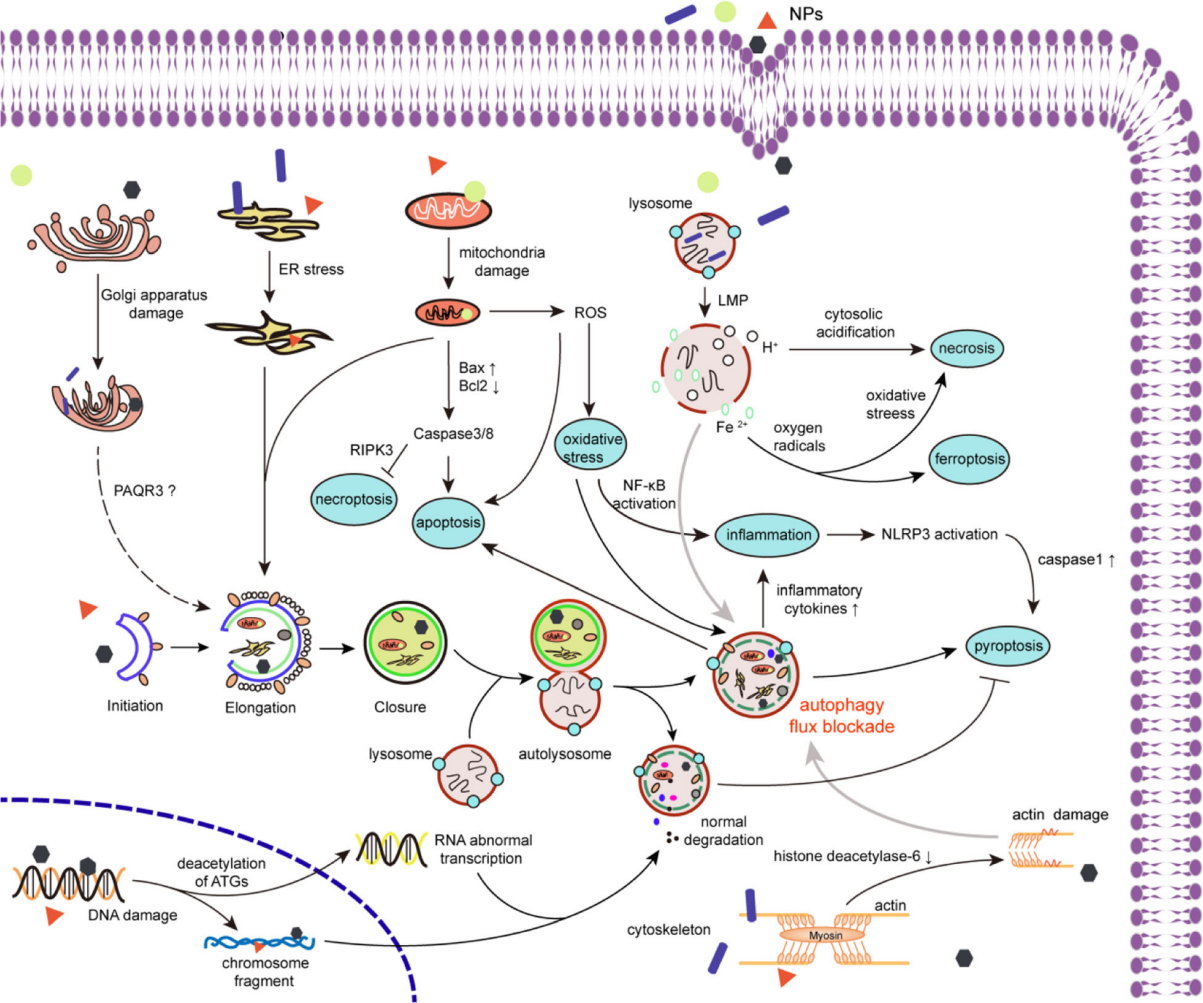
The interactions between NM-mediated autophagy and other biological effects. NMs endocytosed into cells can impaire various organelles including golgi apparatus, endoplasmic reticulum, mitochondria and lysosomes. The damaged organelles are sequestered by primary autophagosomes, which are fused with lysosomes to form autolysosomes, leading to degradation or autophagic flux blockade occur. Autophagy dysfunction will further cause cell inflammation, oxidative stress, apoptosis and pyroptosis. NMs induced LMP can result in cytoplasmic acidification and release of Fe^2+^, which directly relate to autophagic flux blockage and further cell necrosis. The mitophagy dysfunction can lead to caspase 8 activation, which can inhibit the expression of RIPK3, thereby inhibiting necroptosis. NMs cause RNA damage through the deacetylation of autophagy-related proteins and DNA damage through accumulation of chromosome fragments, both activating the autophagy pathway. Impairment of cytoskeleton is mainly manifested by the damage of actin and the decreased expression of histone deacetylase-6, which eventually leads to the disorder of autophagic flux

Ferroptosis is a new form of regulated cell death (RCD) that mainly relies on iron accumulation and lipid peroxidation [29, 197] Furthermore, functionalized NPs have been shown to induce ferroptosis in cancer cells presumably due to pronounced lipid peroxidation and depletion of glutathione [198, 199]. However, a high level of ferroptosis not only kills cancer cells but also causes tissue injury. Considering that induction of autophagy contributes to ferroptosis, how lipid peroxidation affects autophagosome formation or degradation as feedback loops and how NMs play an upstream regulatory role in the autophagy-ferroptosis axis may be directions worth further investigating. Figure 3 shows the intracellular interactions between NM-mediated autophagy and other biological effects.

### Possible therapies related to NM-mediated autophagy

Because autophagy dysfunction plays a key role in the occurrence of diseases, we can utilize NPs that either induce or inhibit autophagy to restore cellular homeostasis in the body and even cure diseases (Table 3). For instance, cytoprotective autophagy has advantageous effects on sick cells. Cytotoxic autophagy can result in the direct clearance of cancerous cells or make them susceptible to chemotherapy drugs, which is beneficial for the treatment of clinical disease. Thus, it is crucial to comprehend the precise mechanism by which the autophagic process is involved in corresponding disorders.

**Table 3:** Possible therapies of NM-mediated autophagy

NPs	Size (nm)	Combined agent	Disease type	Target cells	Dose (ug/mL)	Animal models	Exposure method and time	Administration dose(mg/kg)	Autophagy alteration	Treatment effect	References
UCNPs	–	bare	Atherosclerosis	foam cells	8	–	–	–	induction of autophagy LC3 ↑, beclin 1 ↑, P62 ↓	ROS, enhanced the cholesterol efflux	[38]
SPIO NPs	207 ± 3	bare	Lung cancer	A549 cells	1.75 mg/ml	Female Balb/c mice (4–6 weeks old)	intraperitoneal injection for 30 days	225 μg magnetite equivalent	induction of autophagy	in vitro: elevated ROS level, membrane damage, apoptosis in vivo: induced magnetic hyperthermia and antitumor	[96]
MWCNTs	10 ~ 20	bare	neurovascular disorders	–	–	Wistar rats	intraperitoneal injection for 14 days	2.5	induction of autophagy LC3 ↑, Beclin 1 ↑, P62↓	impaire the congnitive ability of rats	[116]
Acid NPs	102 ± 19	PEG	Liver Cancer	HepG2 cells	50	C57BL/6 J mice (6 weeks old)	intravenous injection for 14 days	10	induction of autophagy LC3 ↑	in vitro: apoptosis, autophagic cell death in vivo: tumor growth inhibition	[168]
SeNPs	~ 240	curcumin	Ehrlich s ascites carcinoma	HCT116 cells	1,2,5,10,20	Female Swiss albino mice			decrease autophagy LC3 ↓, Beclin 1 ↓	In vitro: ROS production, MMP reduction in vivo: inhibition of tumor growth	[170]
Fe_3_O_4_NPs	51.34 ± 14.71	bare	Lung cancer	A549 cells	100	–	–	–	induction of autophagy LC3 ↑	ROS, mitochondrial damage necrosis, autophagic cell death	[200]
Fe_3_O_4_ NPs	162.6 ± 15.6	Chitosan	Stomach cancer	SGC7901, SGC7901/ADR cells	10	nude mice (5–6 weeks old)	intravenous injection for 28 days	5	induction of autophagy LC3 ↑	in vitro: mitochondrial dysfunction, excessive ROS, DNA damage, cell death in vivo: tumor growth inhibition	[201]
ZnO NPs	50	bare	oral cancer	CAL 27 cells	25	–	–	–	induction of autophagy LC3 ↑, Beclin 1 ↑, P62↓	ROS, mitochondrial dysfunction, cell death	[202]
Ag NPs	26.5 ± 8.4	bare	melanoma	HeLa cells	2,5,10,15,20	Male C57BL/ 6 mice (6–8 weeks old)	subcutaneous injection for 4 days	1.5	induction of autophagy autophagosome accumulation	in vitro: inhibition of apoptosis in vivo: promote tumor growth	[203]
CuS NPs	–	bare	Prostate Cancer	RWPE-1 cells	80,120,160	nude male mice (4–8 weeks old)	intraperitoneal injection for 3 weeks	120(μg/ml)	Autophagy blockage LC3 ↑	In vitro: autophagosome accumulation In vivo: inhibit prostate tumor growth with NIR-light	[204]
Lactoferrin nanostructures	137.0 ± 33.65	bare	AD	PC12 cells	10uM	Wistar rats	intraperitoneal injection for 7 days	5	induction of autophagy Atgs ↑, LC3 ↑	in vitro: apoptosis inhibition, neuroprotective effect in vivo: improved spatial memory and learning	[205]
C60 fullerene	–	PEG	AD	Neuro-2A cells	10uM	–	–	–	induction of autophagy LC3 ↑	apoptosis inhibition, cell survival, neuroprotective effect	[206]
CuS NPs	11 ± 2.6	bare	Atherosclerosis	VSMCs	0.4	male mice (6–8 weeks old)	intragastric administration for 12 weeks	10	induction of autophagy LC3 ↑	in vitro: reduced VSMC foam cell formation, enhanced cholesterol efflux in vivo: inhibition of VSMC foam cell formation and attenuation of atherosclerotic lesion in mice	[207]
Fum-PD NPs	252	bare	Arthritis	SEVC4	20 nM	male C57BL/6 mice (6–8 weeks old)	intravenous injection for 9 days	0.3	induction of autophagy LC3 ↑, LAMP-1 ↑, p62↓	in vitro: enhanced NO production, inhibited inflammation in vivo: inflammatory response and inflammatory	[208]

### Anticancer therapy

#### Autophagy preference in cancerous cells

It has been proposed that autophagy activity in tumor cells is higher than in normal cells, which may be related to the higher sensitivity of tumor cells toward oxidative stress [200, 201]. For example, 2-methoxyestradiol (2-ME) could kill leukemia cells rather than normal lymphocytes by causing the production of ROS, an effect that was further confirmed to be associated with excessive autophagy [202]. Compared with normal mouse astrocytes, the treatment of cancer cells with H_2_O_2_ exerted marked autophagy activation and decreased cell viability [203]. Additionally, cancer cells require higher levels of ATP by mitochondria to maintain their rapid proliferation and elevated metabolism. Any mitochondrial damage might therefore result in autophagy induction more rapidly than noncancerous cells [204]. The above data provide a theoretical basis for selective induction of autophagy in cancer cells.

#### Dual role of autophagy in tumor progression

In early-stage tumors (dysplastic stage), autophagy acts as a tumor suppressor to eliminate certain harmful substances and conserve cell homeostasis. For instance, in liver cancer at the dysplastic stage, autophagy was able to slow tumor formation by reducing ROS production, DNA damage, and the release of inflammatory cytokines [204]. Knockout of key autophagy-related genes promoted tumorigenesis, as confirmed by the development of a variety of malignant tumors in Becn1^+/−^ mice. The authors further demonstrated that autophagy could limit the expression of B-cell CLL/lymphoma 10 (BCL10), ultimately inhibiting the formation of an NF-κB signaling pathway-regulated inflammatory microenvironment [183]. At the genetic level, autophagy inhibition can aggravate DNA damage, leading to DNA mutation accumulation and genomic instability, which may contribute to transformation from a normal to a cancerous cell [205].

**Fig. 4:**
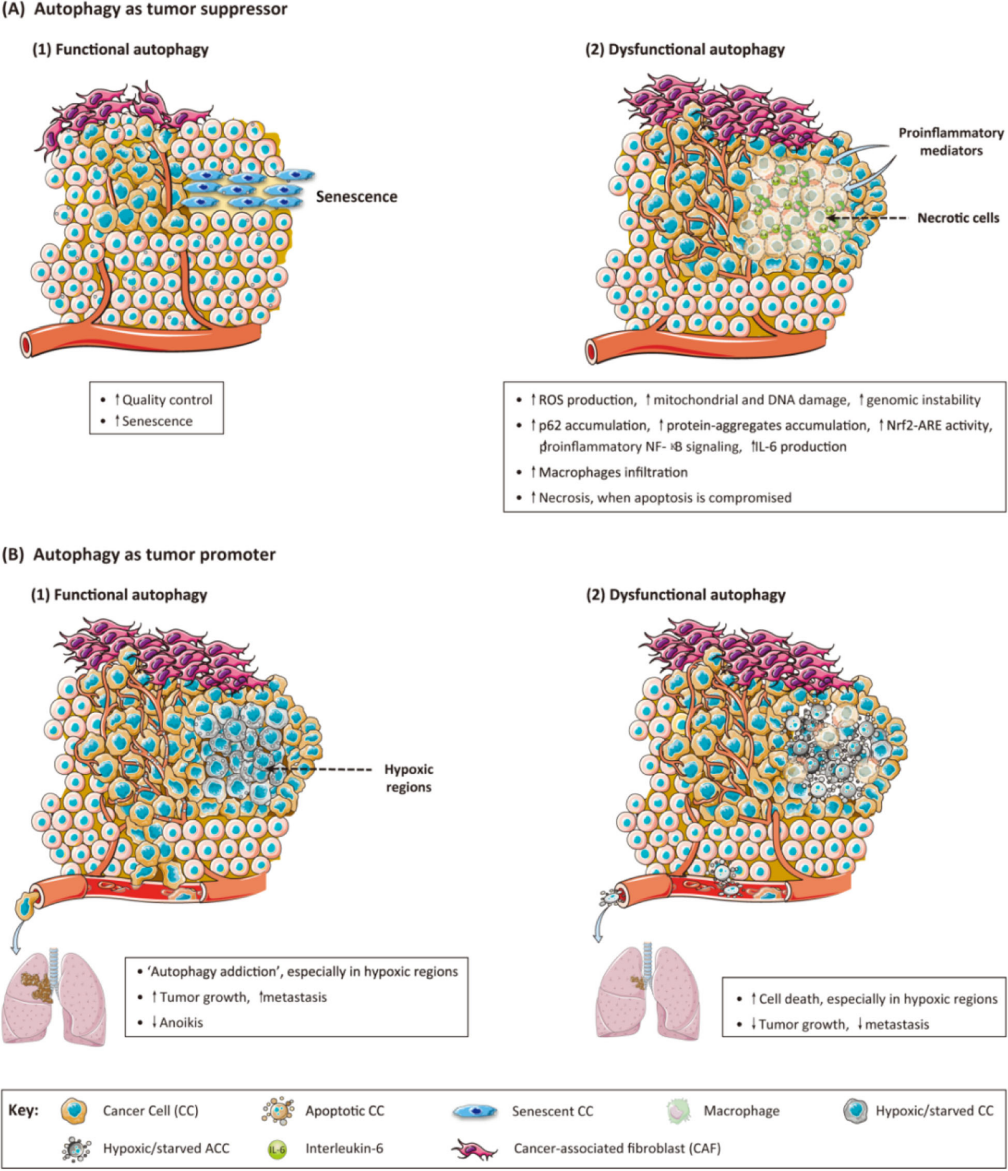
Dual role of cancer cell-associated autophagy in cancer progression. This figure depicts recently described mechanisms supporting a tumor suppressor or tumor promoter role for autophagy. **a** Autophagy as tumor suppressor mechanism. (1) Functional autophagy acts as a quality control mechanism that, under stressed conditions, either restores homeostasis or induces senescence, thus preventing tumorigenesis. (2) Genomic instability, chronic inflammation, p62 accumulation, or increased inflammation associated with tumor initiation and progression under conditions of defective autophagy support a tumor suppressor role for autophagy in cancer progression. **b** Autophagy as tumor promoter mechanism. (1) ‘Autophagy addiction’, especially observed in hypoxic regions of solid tumors, and decreased anoikis help sustain cancer cell viability by promoting malignant growth and metastasis. (2) The increased cancer cell death, especially in hypoxic regions, and reduction of tumor growth and metastasis observed under conditions of autophagy defects indicate that autophagy is a tumor-promoting mechanism in solid tumors [206]. Copyright (2013), with permission from Elsevier

Autophagy may act as a prosurvival mechanism in established tumors, which can degrade damaged proteins and consume ROS [206, 207]. The difficulties in clinical antitumor treatment lie in reducing the autophagic activity of tumor cells and improving the sensitivity of chemotherapy drugs. A study of gastric cancer (GC) cells showed that the autophagy pathway was easily activated through inhibiting PI3K/mTOR expression to resist drug sensitivity [208]. Additionally, increased autophagic flux was beneficial for mammary cancer progression through the promotion of mitophagy and maintenance of redox homeostasis; excessive accumulation of damaged mitochondria and ROS were thus avoided [209]. Consistent with these data, p62 has been shown to support tumor progression through the activation of a variety of transcription factors including Nrf2 [210].. The dual role of autophagy in tumor progression is illustrated in Fig. 4.

#### Selective killing of cancer cells

Autophagy induction as a method in cancer therapy based on the potential benefits of selectively killing cancer cells without (or minimally) impairing noncancerous cells has received increasing attention. For instance, two types of iron oxide NMs can induce autophagy in cancer cells to exert anti-tumor effects, among which ROS were an important intermediate, and there was no significant change in normal cell viability [211, 212]. ZnO NP-treated oral cancer cells (CAL27 cells) showed significant decreases in cell viability via PINK1/Parkin-regulated mitophagy [213]. Another TLR-4/TLR-9 signaling cascade was involved in the GO-induced autophagy process, which inhibited the growth of colon cancer cells (CT26 cells) [214]. These findings suggest that autophagy removes substances needed for tumor cell growth and creates a microenvironment that is not suitable for tumor growth, such as ATP deficiency and a balanced redox system.

When autophagy acts as a prosurvival pathway for cancer cells, the inhibition of autophagy by NMs is beneficial for killing tumor cells. For example, polyethyleneimine (PEI)-coated iron oxide NPs (IONPs) exhibits high uptake in cancer cells, resulting in excessive ROS production and subsequent apoptosis through inhibiting autophagy in a dose-dependent manner [215]. Apart from functionalization of NMs, the addition of the autophagy inhibitor wortmannin or CQ can increase the anticancer effect of AgNPs and copper sulfide (CuS) nanoplates in malignant tumor cells through decreasing autophagy activity [216, 217]. It is worth noting that two research complications must be solved in the future. First, how can we distinguish the different roles of autophagy in tumorigenesis (promoting or inhibiting tumor survival)? Second, how can we control the autophagy level of NMs to avoid damage to normal cells?

#### Treatment of diseases in the CNS

The primary cause of most neurodegenerative diseases, including Alzheimer’s disease (AD) and Parkinson’s disease (PD), is autophagy deficiency, making the targeted induction of autophagy a pivotal aim for their treatment. However, excessive autophagy activation can also lead to nerve damage, and therefore, preserving moderate autophagy levels is essential for maintaining normal nerve function.

#### Autophagy and neurodegenerative disease

AD and PD are characterized by amyloid beta (Aβ) plaque accumulation in the hippocampus/cerebral cortex regions and aberrant α-synuclein proteins in the striatal region, respectively [218]. In these diseases, downregulation of autophagy was thought to prevent the degradation of these aggregates, leading to their significant accumulation within the cell. Komatsu et al. [113] found that mice lacking Atg7 protein showed significant neurodegeneration and behavioral deficits. Similarly, autophagy dysfunction caused by abnormal mTOR signaling pathway was shown to contribute to parkinsonian changes in mice [219]. Considering that pretreatment of mice with the autophagy inducer rapamycin can reverse the above abnormal behavior by reducing the accumulation of abnormal protein plaques [220, 221], it is valuable to exploit NP-induced autophagy to cure neurodegenerative disease.

For example, lactoferrin-conjugated nanostructures have been reported to restore cognitive deficits in rats through autophagy induction and interfere with the apoptotic caspase cascade in rat pheochromocytoma cells (PC12 cells) [222]. Fullerene-based NMs can activate autophagy as a defense mechanism against Aβ-dependent neurotoxic effects, such as decreasing ROS generation and maintaining MMP [223]. In the treatment of PD, PLGA acidic NPs can restore autophagy activity to its basal level by enhancing lysosomal degradation functions such as normalizing lysosomal pH, thereby facilitating the removal of damaged or abnormal proteins [224]. The targets of autophagy defects in each disease are different, so it is necessary to identify the regulatory autophagy targets in different diseases to improve therapeutic effects.

#### Autophagy and nerve injury

Studies have demonstrated that excessive autophagy activation plays an important role in the pathological development of permanent cerebral ischemia and neurotoxicity of iodine [225, 226]. In an in vitro study, after knockdown of LC3 or Atg7, neuritic degeneration caused by axon injury was decreased, whereas the autophagy inducer rapamycin potentiated these effects [227]. In addition, Purkinje cell axons in an animal model of inherited ataxias frequently contained a large number of autophagosomes and autophagosome-like vacuoles, which could result in Purkinje cell degeneration, suggesting neurotoxic effects of excessive autophagy [228]. However, recent studies on the utilization of NMs for the treatment of autophagy-induced nerve injury are still insufficient, and more thorough studies are required.

#### Treatment of cardiovascular disease

Atherosclerosis is a chronic inflammatory cardiovascular disease. Macrophage-derived foam cells are a major component of atherosclerotic plaques, and with the development of the lesion, the center of the atherosclerotic plaque becomes necrotic [229]. Regarding photodynamic therapy (PDT), upconversion fluorescent NPs significantly induce autophagy in foam cells, leading to cholesterol efflux and preventing the maturation of atherosclerotic plaques [38]. A recent study found that CuS NP-treated vascular smooth muscle cells (VSMCs) were able to stimulate Ca^2+^ influx, followed by autophagy activation to impede foam cell formation [230]. From the perspective of blocking downstream autophagic flux, AgNPs can inhibit the differentiation of monocyte-macrophages by impairing lysosomal function, suggesting prevention of the occurrence of earlier cardiovascular disease [231]. Considering the differences in the pathogenesis of atherosclerosis at different developmental stages, a more reliable animal model should be established to systematically analyze the molecular targets associated with NM-mediated autophagy for a better treatment effect.

#### Other potential therapies

In addition to the widespread attention paid to the nano-therapy of cancer and neurodegenerative diseases, there have been increasing reports on the occurrence and development of immune system disorders and osteoarthropathy, which are closely related to autophagy disorders. For example, MWCNTs were able to downregulate autophagy of T and B cells to its basal level, restoring the autoimmune condition and alleviating the pathological progression of systemic lupus erythematosus (SLE) [37]. In comparison, in the treatment of rheumatoid arthritis (RA), fumagillin prodrug (Fum-PD) NPs can promote autophagic flux, followed by inhibition of the NF-κB signaling pathway-dependent inflammatory response [232]. Additionally, based on the significant effectiveness of anti-ROS therapy, dopamine melanin NPs were utilized to further decelerate cartilage degradation through autophagy activation for efficient treatment of osteoarthritis (OA) [233]. Subsequently, the targeted regulation of autophagy by using direct NMs or as drug carriers has introduce huge potential for clinical treatment of related diseases. However, additional work is still required concerning how to reduce the existing adverse effects of NM-induced autophagy.

### Conclusion and outlook (the dual role of NM-mediated autophagy in biomedicine)

This review has summarized research progress addressing potential hazards and beneficial effects of NM-mediated autophagy in biomedicine. First, the wide applications of NMs in biomedicine (including drug delivery systems, tissue engineering, biosensors, and bioimaging system) have led to growing exposure risks of the human body to NMs and their composites. Thus, potential in vivo and in vitro toxic manifestations related to NM-mediated autophagy and mechanisms of action contributing to these adverse effects are discussed. Conversely, it is worth noting that the interactions between autophagy and other biological effects following NM exposure are complicated; thus, NM-mediated autophagy can serve a dual role in biomedicine. Although some of the regulatory process remains unclear, it has shown great potential to exploit NMs to regulate autophagy in the treatment of related clinical diseases, including cancer, neurodegenerative, and cardiovascular diseases. Currently, as autophagy is a multi-factor regulatory process affected by different physicochemical properties of NMs and varying animal or cell models, the data are insufficient to draw conclusions about the influence of NM-regulated autophagy on human health. This review will serve as an important step in understanding the dual role of NM-mediated autophagy and further promoting their beneficial applications in the field of biomedicine.

## Data Availability

Databases/repositories and materials is not applicable in this review.

## Abbreviations

Aβ: Amyloid beta
AD: Alzheimer’s disease
AgNPs: Silver NPs
ALT: Alanine aminotransferase
AST: Aspartate aminotransferase
Atg: Autophagy-related protein
AuNPs: Gold NPs
A549 cells: Human lung adenocarcinoma cells
Bcl-2: B-cell lymphoma 2
BCL10: B-cell CLL/lymphoma 10
CAL27 cells: Oral cancer cells
CeO_2_: Cerium dioxide
CME: Clathrin-mediated endocytosis
CNTs: Carbon nanotubes
CoCr: Cobalt and Chromium
COOH-PS: Carboxylated polystyrene
CQ: Chloroquine
CTAB: Cetyl trimethylammonium bromide
CT26 cells: Colon cancer cells
CuO: Copper oxide
CuS: Copper sulfide
CvME: Caveolae-mediated endocytosis
ER: Endoplasmic reticulum
ERS: Endoplasmic reticulum stress
Fe_2_O_3_: Iron oxide
Fe_3_O_4_: Ferrosoferric oxide
Fum-PD: Fumagillin prodrug
GC: Gastric cancer
GCNF: Graphite carbon nanofibers
GO: Graphene oxide
GO-CQ: Graphene oxide-chloroquine
GONPs: GO nanoplatelets
HCT 116 cells: Human colon cancer cells
HE: Hematoxylin and eosin
HepG2 cells: Human hepatoma cells
HIF-1: Hypoxia-inducible factor 1
HL-60 cells: Human promyelocytic leukemia cells
HUVECs: Human umbilical vein endothelial cells
IF: Intermediate fibers
IL-6: Interleukin-6
IONPs: Iron oxide NPs
JNK: c-Ju Au NPs inal kinase
LC3: Microtubule-associated protein light chain 3
LMP: Lysosomal membrane permeabilization
LTP: Long-term potentiation
MCF7 cells: Human breast ccancer cells
MF: Microfilaments
M-FeNPs: Magnetic iron oxide NPs
miRNAs: MicroRNAs
MMP: Mitochondrial membrane potential
MSNs: Mesoporous silica NMs
MT: Microtubules
mtDNA: Mitochondrial DNA
MTOR: Mammalian target of rapamycin
mtROS: Mitochondrial reactive oxygen species
NAC: N-acetyl L-cysteine
NH_2_-PS: NH_2_-labeled polystyrene
NMs: Nanomaterials
NPs: Nanoparticles
OA: Osteoarthritis
oMWCNTs: Oxidized multiwalled carbon nanotubes
PAMAM: Polyamidoamine dendrimers
PC12 cells: Rat pheochromocytoma cells
PD: Parkinson’s disease
PDT: Photodynamic therapy
PE: Phosphatidyl ethanolamine
PEG: polyethylene glycol
PEI: Polyethylenimine
PLGA: Poly lactic-co-glycolic acid
PTR: Pleurotus tuber-regium
PU NPs: polyurethane NPs
RA: Rheumatoid arthritis
RCD: Regulated cell death
RES: Reticuloendothelial system
RIP: Receptor interaction protein
ROS: Reactive oxygen species
Se-Cur NPs: Curcumin-loaded selenium NPs
SeNPs: Selenium NPs
SiNPs: Silica NPs
SLE: Systemic lupus erythematosus
SPIONs: Superparamagnetic iron oxide NPs
SWCNTs: Single-walled carbon nanotube
TiO_2_: Titanium dioxide
TLR-4: Toll-like receptor-4
TNF α: Tumor necrosis factor-α
UCNs: Upconversion NPs
UPR: Unfolded protein response
VEGFR2: Vascular endothelial growth factor receptor 2
VSMCs: Vascular smooth muscle cells
ZnO: Zinc oxide
3-MA: 3-methyladenine
2-ME: 2-methoxyestradiol
